# Husband involvement in postnatal care services utilization and associated factors in Bishoftu Town, Central Ethiopia; community-based cross-sectional study

**DOI:** 10.3389/fgwh.2024.1423439

**Published:** 2024-12-13

**Authors:** Hana Israel Gesisa, Befekadu Tesfaye Oyato, Warkitu Sileshi, Husen Zakir Abasimel, Dursa Hussien

**Affiliations:** ^1^ Department of Midwifery, Institute of Health Sciences, Jimma University, Jimma, Ethiopia; ^2^ Department of Midwifery, College of Health Sciences, Salale University, Fitche, Ethiopia; ^3^ Department of Public Health, Kuyu General Hospital, Gerba Guracha, Ethiopia

**Keywords:** husband, postnatal care services, husbands' involvement, Bishoftu, Ethiopia

## Abstract

**Background:**

Both mothers and newborns go through a critical time in their lives during the postnatal period, and the majority of deaths of mothers and neonates happen during this period. Postnatal care is the care given to women and their neonates, starting from delivery to 42 postnatal days. Although the significance of postnatal care in maternal and child health is acknowledged, the influence of husbands in either facilitating or impeding access to these services has been largely unexplored. So it's important to find out husbands’ involvement in postnatal care service utilization in Bishoftu town.

**Objectives:**

To assess husband involvement in postnatal care services utilization and associated factors in Bishoftu town, Central Ethiopia.

**Methods:**

A community-based cross-sectional study was conducted in Bishoftu town, central Ethiopia, between April 1 and 27, 2022, among 624 fathers. After being selected through simple random sampling, 610 husbands were interviewed face-to-face. The data was then entered into Epi-Data version 3.1 and analyzed using SPSS version 26. Binary logistic regression was employed for analysis and variables with a *p*-value ≤0.2 in the bivariate analysis were entered into multivariable logistic regression analysis. In multivariable analysis, statistical significance was declared at *p*-value <0.05.

**Results:**

This study reveals that overall husband involvement in postnatal care utilization was found to be 34.1% with a 95% CI (30.3%–38.0%). Residing in urban areas (AOR = 2.3, 95% CI 1.39–3.82), having good knowledge of neonatal (AOR = 3.1, 95% CI 2.04–4.7) and maternal danger signs and complications during the postnatal period (AOR = 2.44; 95% CI: 1.64, 3.63), having history of child illness (AOR = 2.18; 95% CI: 1.4–3.3), and accompanying wife in antenatal care services (AOR = 2.73, 95% CI 1.82–4.07) were the factors found to determine husband's involvement in postnatal care service utilization.

**Conclusion:**

The level of husband involvement in their spouse's postnatal care service utilization was relatively low in this study. Encouraging husbands to participate in prenatal care services, availing healthcare facilities to rural communities, and increasing community awareness of maternal and neonatal warning signs might boost husbands’ involvement in postnatal care services utilization.

## Introduction

Global health systems face persistent and significant challenges in reducing maternal and neonatal mortality rates. The World Health Organization (WHO) reports that each year, around 287,000 women die, equivalent to nearly 800 deaths per day, due to complications arising from pregnancy, labor and delivery, and the postpartum period worldwide (1). In contrast, 2.6 million infants die within their first month of life every year, with 7,000 newborns dying each day (2).

Nearly all maternal and neonatal deaths occur in developing countries. In 2020, Sub-Saharan Africa reported 500 maternal deaths per 100,000 live births, accounting for around 70% of global maternal deaths, followed by Central and Southern Asia, which contributed nearly 17%. Neonatal mortality was also highest in sub-Saharan Africa and southern Asia, with rates of 27 and 23 deaths per 1,000 live births, respectively, in 2020 (1–3). According to the 2016 Ethiopian Demographic Health Survey (EDHS), the maternal mortality ratio (MMR) was estimated at 412 per 100,000 live births. Additionally, neonatal mortality increased slightly from 29 to 30 deaths per 1,000 live births between 2016 and 2019 (4).

Unfortunately, the majority (62%) of these deaths occur during the postpartum period, and most are preventable (1, 2). An effective strategy to address both maternal and child health related complication is the use of maternal health care services, including postnatal care. Studies have shown that providing women with access to maternal healthcare services, such as PNC, could prevent approximately 80% of maternal deaths (5). However, the utilization of postnatal care services remains low in Sub-Saharan countries, including Ethiopia (4, 6). One of the major barriers preventing women from accessing maternal healthcare services, like postnatal care services, is the lack of male partner involvement (7–9).

Husbands’ involvement in maternal, newborn, and child health (MNCH) can be defined as men actively participating in safeguarding and promoting the well-being of their wives and children (10). Evidence shows that men's involvement in maternal and child healthcare services offers several advantages. For instance, it can facilitate timely access to antenatal and postnatal care services, improve newborn survival, and enhance adherence to recommended postnatal practices (11, 12). Furthermore, supportive husbands play a key role in improving mental health of the mothers by providing emotional support, which is especially important in mitigating postnatal depression (13).

Considering these benefits, the 1994 International Conference on Population and Development (ICPD) in Cairo was the first to emphasize the importance of male involvement in sexual and reproductive health and in prenatal, maternal, and child health (14). In 2015, the World Health Organization (WHO) recommended leveraging maternal, newborn, and child health (MNCH) initiatives to enhance men's involvement during pregnancy, labor, and the postnatal period, aiming to improve maternal and child health outcomes (15). Developing countries like Ethiopia have also implemented various strategies to improve this condition, including health extension programs, couples counseling, and mass media campaigns highlighting the importance of male involvement (16). Although these strategies are effective in increasing male participation, their implementation remains inadequate in countries with high maternal and newborn mortality rates, such as Ethiopia (8).

Findings from various studies show that husbands’ involvement in postnatal care utilization is generally low. For instance, a study in Nepal reported that only 33.8% of male partners were involved in their spouses’ PNC service use (17). Other studies from Africa have shown that 20%, 59.3%, and 87.7% of male partners were involved in their spouse's utilization of PNC services in Ghana, Tanzania, and Nigeria, respectively (18–20). Additionally, a study conducted in the Motta district of Ethiopia reported the lowest rate of husband involvement in PNC utilization (20.8%) compared to other African countries (21). Several factors influence the husband's involvement in postnatal care, including residency, educational attainment, occupation, family type, income, knowledge about neonatal and postpartum danger signs, the spouse's involvement in decision-making, and proximity to medical facilities (17–21).

In countries like Ethiopia, where Islam and Christianity are the predominant religions, patriarchy influences societal views on fatherhood and the roles men play (22). Men are often recognized as primary decision-makers, and in many cases, husbands or partners make health care decisions for women and children without consulting them (23–27). Existing evidence shows that women who are under the influence of their male partners were 21% less likely to utilize PNC services compared to their counterparts (28). Additionally, there is a belief that maternal and child health is solely a woman's issue (8).

Considering the diverse perspectives in Ethiopia, it is crucial to assess the extent of male partners’ engagement in PNC services to reduce delays in seeking health care and thereby decrease maternal and neonatal mortality. While few studies in Ethiopia focus on antenatal care, prevention of Mother to Child Transmission and Birth preparedness and complication readiness, little is known about the level of husband involvement in postnatal care services across different settings. Therefore, the goal of this study was to determine husband involvement in PNC service utilization and to identify factors influencing it in Bishoftu town, Central Ethiopia.

## Methods

### Study setting

A community-based cross-sectional study was conducted from April 1 to 27, 2022. The study took place in Bishoftu, a town in the Oromia region, located 47 kilometers east of Addis Ababa, the capital city of Ethiopia. The town has fourteen kebeles (a small administrative unit), five rural, and nine urban. There were two public hospitals, five health centers, two private hospitals, and ten private clinics providing a range of medical services for the community. According to a 2022 report from the Bishoftu town health office, the town had a total population of 234,970 and 48,972 households (29).

### Population

#### Source population

All husbands whose wives gave birth in the last six months in Bishoftu town.

#### Study population

Husbands whose wives gave birth in the last six months in randomly selected kebeles of Bishoftu town were the study population.

#### Eligibility criteria

This study included all husbands who had children less than six months of age and had lived in Bishoftu town for a minimum of six months.

#### Sample size determination

The sample size for this study was calculated using a single population proportion formula with the following assumptions: Z*α*/2 = 1.96, a margin of error of 0.04, and a prevalence rate of 20.8%, which is derived from a study conducted in the Motta district on husband involvement in postnatal care service utilization and associated factors (21).

*p* = proportion of male partner involved in PNC care, which was 20.8%, and q = 1-p.

$$\mathrm{Sample}\;\mathrm{size}=\frac{{\left(1.96\right)}^{2}0.208\;(1-0.208)}{0.04^{2}}=396$$

By considering the design effect of 1.5% and 5% non-response rates, the minimum sample size needed for this study was determined to be 624.

#### Sampling technique and procedure

A two-stage random sampling method was used to draw the sample respondents. In the first stage, 5 kebeles were selected using simple random sampling techniques from a total of 14 kebeles. 3 kebeles were from urban and 2 kebeles were from rural areas proportionally.

Lists of households with husbands who had children less than 6 months of age within the selected kebeles were extracted from the family folder of the health extension workers. The total sample size was then proportionally allocated to the selected kebeles based on the number of households with husbands whose wives had given birth in the last six months in their respective kebeles. A simple random sampling technique (computer-generated list of random numbers) was used to select the final sample size (624) from the list of households with husbands whose wives had given birth in the last six months.

### Study variables

#### Dependent variable

Husband involvement in postnatal care services utilization.

#### Independent variables

Socio-demographic factors like age of husband, age of recent child, educational status and occupation of the husband and wife, religion, residence, type of family, and average monthly income were independent variables of this study. Obstetrics-related characteristics of the respondents’ wives included number of children, place of birth of the last baby born, mode of delivery, wife antenatal care visit, antenatal care (ANC) visits accompanied by the husband and frequency, visit at postnatal care (PNC) unit after delivery, history of infant illness, place of seeking care, decision maker to seek care for the baby, history of child death, and time required to reach the nearest healthcare facility. Additionally, husbands’ knowledge of neonatal and maternal danger signs were included as an independent factors in this study (17–21).

#### Operational definition and measurements

The outcome variable for this study (husband involvement in postnatal care services utilization) was binary and was coded as “1” if husband involved in wife's postnatal care utilization and “0” otherwise. It was assessed using a ten-point index which comprises: discussing PNC services and complications occurring in the postpartum period with health professionals, discussing with a spouse regarding PNC service, accompanying a spouse for PNC service, providing physical support, discussing postpartum family planning methods, providing emotional support, joint decision making power with the spouse on PNC service, providing financial support, helping with domestic tasks, and taking care of children.

Each of the ten items was assigned a score of one (1) if the participant performed the activity and zero (0) if it was not performed. In order to categorize participants as either involved or not involved a total score was generated and 50% was utilized as the cut-off point.

Husband involved in PNC services utilization: a husband who scored 50% or more of the total score.

Husband not involved in PNC services utilization: a husband who scored less than 50% from the total score.

Husband accompanied in PNC services: A husband who accompanied his wife to the PNC clinic for at least one follow-up visit, excluding the initial 6-hour PNC visit (30).

Neonatal danger signs: signs that indicate abnormal health conditions and that occur during the first 28 days of life (31).

Knowledge of neonatal danger signs: husbands’ level of awareness or mindfulness about neonatal danger signs (32).

Good knowledge: husbands who were able to mention at least three neonatal danger signs among the 12 neonatal danger signs without prompt (31).

Knowledge of obstetrics danger signs during postpartum period: In this study, a husband who could mention at least three obstetric danger signs, with or without prompting, is considered knowledgeable. Prompting was done by giving the spouse additional time that would help them recall what their wives might have experienced in the postnatal period (33).

### Data collection tools and techniques

After reviewing pertinent and related literature (17–21, 29), a semi-structured questionnaire was prepared in English and translated to Afan Oromo and Amharic. To maintain consistency, it was then professionally back-translated into English. The questionnaire was given in both Amharic and Afan Oromo, depending on the participants’ preferred language to facilitate understanding.

Data were collected by seven trained bilingual bachelor degree holders in nursing, under the supervision of health extension workers. The data collection was conducted through face-to-face interviews using a semi-structured questionnaire during home visits. It was supervised by three experienced supervisors. The data were collected whenever the husbands were available, including during weekends and lunchtime. Additional visits were made if study households were closed or respondents were not present at the time of data collection.

### Data quality control

A properly designed instrument was developed after reviewing different literature and then pre-tested on 5% of the sample size in a comparable setting in the town of Adama prior to the actual survey to check whether the questionnaire was simple and easily understandable. Two-day training was given for data collectors and supervisors on the instrument. The collected data were checked out every day by the principal investigator for consistency and completeness. Problems encountered were reported to supervisors and the principal investigator for immediate action.

### Data processing and analysis

After being manually checked for completeness and consistency, the data were coded, entered into Epi-Data V.3.1, and exported to SPSS V.26 for further analysis. Cross tabulation was done to clean the missing values, explore the data, and determine the expected count per cell. Bivariate and multivariable logistic regression models were used to identify the existence of association between outcome and explanatory variables.

Variables with a *p*-value <0.2 in the bivariate analysis were considered candidates for multivariable logistic regression, in which statistical significance between dependent and independent variables was determined at a *p*-value <0.05. Adjusted odds ratio (AOR) at a 95% confidence interval (CI) was used to show the degree to which the independent variables explained the dependent variable. Multicollinearity was checked by using variance inflation factor (VIF), and tolerance test, which were 4.5 and 0.22, respectively. The fitness of the model was tested using the Hosmer-Lemeshow test, and the value was 0.955. Descriptive statistics were used to determine the frequency of different variables. The result of the study was then presented using simple frequencies, figures and tables.

## Results

### Socio-demographic characteristics of participants and their wives

This study involved a total of 610 participants, yielding a response rate of 98.2%. The mean age of the respondents was 34.35 years (SD ± 6.509), with ages varying from 22 to 49 years. Fifty-seven percent of the participants lived in urban areas, and just over half of the respondents (53.6%) were Orthodox.

Regarding their educational and occupational status, more than six out of ten respondents (64.8%) had completed their secondary education, two-fifths were government employees, and fifty-three percent of the study participants had female children (Table 1).

**Table 1:** Socio-demographic characteristics of respondents and respondents’ wives who gave birth in the last 6 months in Bishoftu town, Central Ethiopia, 2022 (*n* = 610).

Variables	Category	Frequency	Percentage (%)
Age of husband	20–24	44	7.2
	25–29	132	21.6
	30–34	124	20.3
	≥35	310	50.8
Age of the child in days	0–28	81	13.3
	>28	529	86.7
Religion	Protestant	148	24.3
	Orthodox	326	53.4
	Catholic	34	5.6
	Muslim	69	11.3
	Wakefeta	33	5.4
Husbands’ educational level	No formal education	43	7
	Primary education	172	28.2
	Secondary and above	395	64.8
Husbands’ occupation	Government employee	247	40.5
	Private employee	149	24.4
	Self-employed & daily labourer	139	22.8
	Farmer	75	12.3
Wives’ educational level	No formal education	52	8.5
	Primary education	167	27.4
	Secondary and above	391	64.1
Wives’ occupation	Government employee	83	13.6
	Private employee	109	17.9
	Self-employed & daily labourer	158	25.9
	Farmer	15	2.5
	Housewife	245	40.2
Family type	Nuclear	480	78.7
	Joint	130	21.3
Average monthly income	<=4,950	298	48.9
	>4,950	312	51.1

### Obstetrics-related characteristics of the respondents’ wives

Nearly three-quarters of the respondents’ wives (72%) had given birth at least twice, and over half (56.9%) had delivered their babies in a hospital. Nearly all wives (97.4%) reported attending ANC during their pregnancy. Of the respondents, forty-eight percent accompanied their wives to ANC visits. Among them, just 1% attended all four visits, while over half (57.5%) accompanied their wives just once.

Seven in ten participants (73.6%) visited their wives at the postnatal care unit within the first 6 h, and thirty-one percent accompanied their wives during discharge from the unit (Table 2).

**Table 2:** Obstetric characteristics of respondents’ wives who gave birth in the last 6 months in Bishoftu town, Central Ethiopia, 2022 (*n* = 610).

Variables	Category	Frequency	Percentage (%)
Number of children	1	171	28
	2	177	29
	>=3	262	43
Place of delivery of the last baby born	Health center	263	43.1
	Hospital	347	56.9
Mode of delivery	SVD	435	71.3
	Instrumental	109	17.9
	CS	66	10.8
Accompany wife during PNC discharge after delivery	Yes	191	31.3
	No	419	68.7
History of last baby sick	Yes	189	31
	No	421	69
Place of seeking care (*n* = 189)	Health center	83	43.9
	Hospital	104	55
	Traditional	2	1.1
Decision-maker to seek care (*n* = 189)	Husband	75	39.7
	Wife	29	15.34
	Wife's relative	4	2.1
	Husband and wife	81	42.86
Wife's ANC follow up	Yes	594	97.4
	No	16	2.6
Frequency of ANC (*n* = 594)	One times	6	1
	Twice	15	2.52
	Three times	152	25.6
	Four times and above	421	70.88
Accompanied by the husband during a visit	Yes	292	47.9
	No	318	52.1
History of child death	Yes	5	0.8
	No	605	99.2
Time to reach the closest healthcare institution	<=30 min	355	58.2
	>30 min	255	41.8

Notes: ANC, antenatal care; CS, cesarean section; SVD, spontaneous vaginal delivery.

### Husbands’ knowledge about neonatal and maternal danger signs and complications

**Figure 1:**
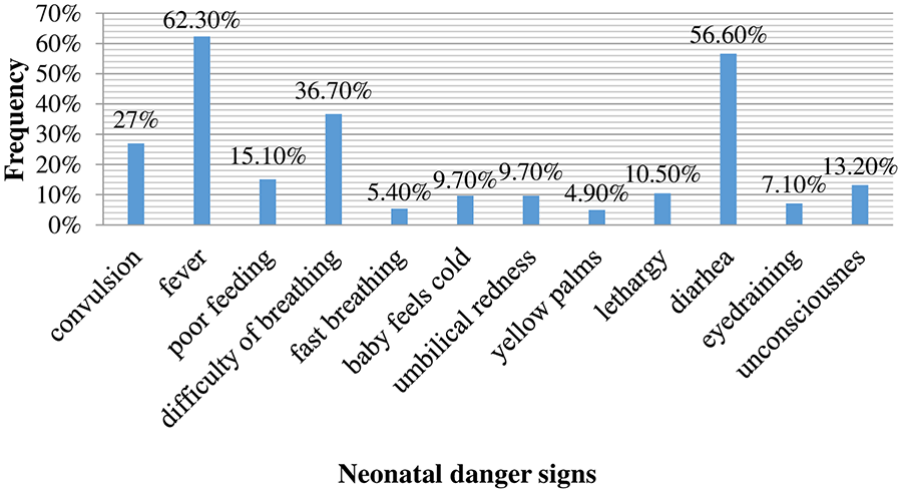
Proportion of husbands who mentioned the key neonatal danger signs in Bishoftu town, Central Ethiopia, 2022 (*n* = 610).

Forty-four percent of husbands (44.3%) had good knowledge of neonatal danger signs. The most commonly mentioned dangers signs among the respondents were fever (62.1%) and diarrhea (56.6%) (Figure 1).

Of the total respondents, 43.6% of husbands had good knowledge of maternal danger signs and complications that occur during the postnatal period. 263 husbands (43.1%) identified fever as a danger sign, while only 87 husbands (14.3%) mentioned Calf muscle pain, redness, and swelling as danger signs (Table 3).

**Table 3:** Husbands’ knowledge of maternal danger signs and complications during the postpartum period in bishoftu town, central Ethiopia, 2022 (*n* = 610).

Maternal danger signs and complications	Frequency	Percentage (%)
Fever	263	43.1
Severe headache	169	27.7
Heavy vaginal bleeding	132	21.6
Foul smelling vaginal discharge	113	18.5
Blurring of vision	143	23.4
Calf muscle pain, redness and swelling	87	14.3
Severe weakness	306	50.2
Pregnancy-induced hypertension	103	16.9
Uterine/breast infection	104	17
DVT	90	14.8
Postpartum depression	98	16.1
Wound infection	118	19.3

Note: DVT, deep vein thrombosis.

### Husbands’ involvement in PNC service utilization

In this study, overall husband involvement in postnatal care utilization was found to be 34.1% with a 95% CI (30.3–38.0) (Table 4).

**Table 4:** Husband involvement in PNC service utilization in Bishoftu town, central Ethiopia, 2020 (*n* = 610).

Components		Frequency	Percentage (%)
Discusses on PNC service with their partner	Yes	200	32.8
	No	410	67.2
Shared decision-making powers on PNC with wife	Yes	202	33.1
	No	408	66.9
Accompanies partner to the health care facility	Yes	123	20.2
	No	487	79.8
Discusses on family planning with their partner	Yes	298	48.9
	No	312	51.1
Discusses on PNC service and complications during the postpartum period with her health care provider	Yes	234	38.4
	No	376	61.6
Provides physical support to his partner during postnatal period	Yes	232	38
	No	378	62
Provides emotional support to their partner (encourage) for PNC service utilization	Yes	294	48.2
	No	316	51.8
Provides financial support to their partner for PNC service utilization	Yes	214	35.1
	No	396	64.9
Helps domestic tasks	Yes	214	35.1
	No	396	64.9
Looks after children	Yes	202	33.1
	No	408	66.9

Notes: PNC, postnatal care.

### Factors associated with husbands’ involvement in PNC service utilization

In the bi-variable logistic regression analysis, factors such as husband's level of education, residence, accompanying the wife during the ANC visit, visiting the wife during the first 6 h of PNC, knowledge of neonatal and maternal danger signs and complications, history of child illness, and the time taken to reach the nearest health care facility were candidate variables for multivariable analysis.

Place of residence, accompanying the wife during the ANC visit, knowledge of neonatal and maternal danger signs and history of child sickness were independently associated with husband involvement in postnatal care service utilization in multivariable logistic regression analysis (Table 5).

**Table 5:** Bi-variable and multivariable logistic regression of factors associated with husbands’ involvement in PNC service utilization in bishoftu town, central Ethiopia, 2022 (*n* = 610).

Variables	Categories	Husband involvement		COR (95% CI)	AOR (95% CI)
		Involved	Not involved		
Level of education	No formal education	7	36	1	1
	Primary	49	123	2.05 (0.85, 4.91)	1.50 (0.59, 4.22)
	Secondary & above	152	243	3.22 (1.30, 7.40)	1.06 (0.40, 2.80)
Residence	Rural	56	204	1	1
	Urban	152	198	2.7 (1.94, 4.02)	2.30 (1.39, 3.82)*
Husband accompanied wife during ANC visit	No	66	252	1	1
	Yes	142	150	3.62 (2.53, 5.16)	2.73 (1.82, 4.07)*
Visited wife during the first 6 h of PNC	No	61	100	1	1
	Yes	147	302	0.79 (0.55, 1.16)	0.70 (0.48, 1.16)
Knowledge of neonatal danger signs	Poor	67	273	1	1
	Good	141	129	4.45 (3.11, 6.37)	3.10 (2.04, 4.70)*
Knowledge of maternal danger signs and complications	Poor	78	266	1	1
	Good	130	136	3.26 (2.30, 4.62)	2.44 (1.64, 3.63)*
History of child sickness	No	126	294	1	1
	Yes	82	108	1.77 (1.24, 2.53)	2.18 (1.40, 3.30)*
Time to reach the nearest health facility	>30 min.	74	181	1	1
	<=30 min.	134	221	1.48 (1.05, 2.09)	0.83 (0.52, 1.33)

Notes: *p value <0.001; PNC: postnatal care; ANC: antenatal care.

Husbands who reside in urban places were twice as likely (AOR = 2.3, 95% CI 1.39–3.82) to be involved in their spouse's utilization of PNC services as husbands who live in rural areas. Similarly, husbands who accompanied their wives during the ANC visit were 2.7 times as likely to be involved in PNC service utilization compared to their counterparts (AOR = 2.73, 95% CI 1.82–4.07).

Husbands who had good knowledge of neonatal and maternal danger signs and complications were 3 times (AOR = 3.1, 95% CI 2.04–4.7) and 2 times (AOR = 2.44, 95% CI 1.64–3.63) more likely to be involved in their spouse's utilization of PNC services than their counterparts. Husbands with a history of child illness were twice as likely to be involved in their spouse's utilization of PNC service compared to those without such a history (AOR = 2.18, 95% CI 1.4–3.3) (Table 5).

## Discussion

This study investigated husband involvement in PNC service utilization and associated factors in Bishoftu town, Central Ethiopia. Consequently, the findings revealed that 34.1% of husbands were involved in their spouse's PNC service utilization. The husband's involvement in their spouse's usage of PNC services was significantly influenced by their place of residency, knowledge of maternal and neonatal danger signs, accompaniment to the ANC service, and history of child illness.

The overall level of husband involvement in PNC service utilization was 34.1% (95% CI: 30.3–38). This finding aligns with the study conducted in Nepal, which reported a rate of 33.8% (17). The similarity may be attributed to comparable educational statuses in both settings.

Our findings were higher compared to the studies carried out in western Ghana (20%) (18) and Motta Ethiopia (20.8%) (21). A possible explanation for this difference could be that our study had a higher number of study participants compared to the study conducted in western Ghana. Additionally, our study included a higher proportion of participants from urban areas, whereas the study from Motta, Ethiopia, had a higher percentage of respondents from rural areas, with more than half lacking formal education. Husbands residing in urban areas and having formal education may be more likely to be involved in their spouses’ PNC utilization compared to their counterparts. However, this finding was lower than the findings in Tanzania (59.3%) (19) and Nigeria (87.7%) (20). The discrepancy might be that the study from Nigeria included urban residents only, whereas our study included both rural and urban areas. Additionally, the study in Tanzania had a large sample size compared to ours.

This study found that residence was a significant factor influencing the husband's involvement in PNC service utilization. Men who lived in urban areas had twice the odds of being involved in their wives’ PNC utilization. This finding is consistent with a study conducted in Motta District, Ethiopia (21). This might be because husbands living in urban areas are more likely to have easy access to health facilities and information regarding postnatal care from various sources. However, surprisingly, this finding contrasts with a study conducted in Tanzania (19), which reported that urban residents had a two-fold lower likelihood of involvement in PNC service utilization.

This study revealed that husbands who had good knowledge of maternal and neonatal danger signs had a greater likelihood of being involved in their spouse's utilization of PNC services than their counterparts. This is in line with the study conducted in Motta, Bangladesh, and Uganda (21, 34, 35). This could be because recognizing danger signs enhances anticipation, which in turn helps minimize the impact of postpartum complications through early healthcare seeking (36, 37).

This study established a statistically significant positive relationship between a history of child illness and husband involvement in PNC service utilization. Male partners who have a history of child illness were 2.18 times more likely to be involved in their spouse's utilization of PNC services. A qualitative study from Tanzania revealed that men took responsibility for making travel arrangements and accompanying their spouses to health care services, particularly for non-routine care (childbirth, emergency care, referral-level care, or a serious illness) (23). This could be because the fear of losing their child drives them to actively engage in postnatal care services.

Husbands who accompanied their wives to antenatal care visits were more likely to be involved in postnatal care service utilization. This might be because participating in antenatal care services allows husbands to gain knowledge about childbirth preparation and the advantages of giving birth in a health care institution. They also basically gain knowledge about the significance of postnatal care services in combination with the linkage of maternal health care services (38, 39). A study from Myanmar showed that, although not significant, mothers who attended the ANC with their partners had a greater chance of utilizing PNC services.

### Strength and limitations of the study

This study is representative of the source population since it was conducted at the community level including both urban and rural areas. The probability sampling method strengthens the representativeness of the study population. Due to the cross-sectional study design nature which assess exposure and outcome at the same point in time the results might not indicate reverse causality. Moreover, participants might also be influenced by social desirability biases.

### Implications for policy, practice, and research

The findings of this study emphasize the importance of encouraging husbands’ involvement in postnatal service utilization to enhance the timely use of facility-based care for obstetric and neonatal problems. To support this, policy initiatives at the national and subnational levels should continue to prioritize the dissemination of culturally appropriate information, educational interventions, and materials regarding maternal and neonatal danger signs and complications, particularly for rural communities. Healthcare practitioners should also consider inviting male partners to attend their wives’ antenatal care sessions as an effective way to reach and educate them about maternal and neonatal danger signs and the importance of utilizing postnatal care for better maternal and neonatal health outcomes. Additionally, future research should focus on conducting qualitative studies to explore and comprehend the influence of socio-cultural gender norms on male partners’ roles in their spouse's utilization of PNC services.

## Conclusions

In this study, husbands’ involvement in their spouses’ postnatal care service utilization was relatively low. Living in an urban area, having a history of child illness, being knowledgeable about maternal and neonatal danger signs and complications during the postnatal period, and accompanying the wife to prenatal care visits were the factors found to determine the husband's involvement in postnatal care services utilization. The results of the study indicated that encouraging husbands to participate in antenatal care services, availing healthcare facilities to rural communities, and increasing community awareness of maternal and neonatal warning signs could all be effective strategies for increasing husbands’ involvement in postnatal care services utilization. Raising community awareness in rural areas through a variety of media, including radio, television, and campaigns, could improve husbands’ involvement in PNC service utilization. Future qualitative research is advised to better understand and explore the impacts of societal gender norms.

## Data Availability

The raw data supporting the conclusions of this article will be made available by the authors, without undue reservation.
